# The Plant Salicylic Acid Signalling Pathway Regulates the Infection of a Biotrophic Pathogen in Grasses Associated with an *Epichloë* Endophyte

**DOI:** 10.3390/jof7080633

**Published:** 2021-08-04

**Authors:** Ming-Zhu Kou, Daniel A. Bastías, Michael J. Christensen, Rui Zhong, Zhi-Biao Nan, Xing-Xu Zhang

**Affiliations:** 1 State Key Laboratory of Grassland Agro-Ecosystems, College of Pastoral Agriculture Science and Technology, Lanzhou University, Lanzhou 730020, China; koumzh18@lzu.edu.cn (M.-Z.K.); zhongr12@lzu.edu.cn (R.Z.); zhibiao@lzu.edu.cn (Z.-B.N.); 2 Resilient Agriculture Innovation Centre of Excellence, AgResearch Limited, Grasslands Research Centre, Palmerston North 4442, New Zealand; Daniel.Bastias@agresearch.co.nz (D.A.B.); mchristensenpn4410@gmail.com (M.J.C.)

**Keywords:** plant defences, *Epichloë* endophytes, fungal pathogens, plant hormones, salicylic acid, jasmonates, symbiosis

## Abstract

The study of the contribution of the plant defence hormones, salicylic acid (SA) and jasmonic acid (JA), in the resistance against pathogens of plants associated with *Epichloë* fungal endophytes has been scanty. We hypothesised that *Epichloë* spp., capable of inducing host plant SA-dependent defences, would increase the levels of plant resistance against biotrophic pathogens. Plants of *Achnatherum inebrians*, with and without the fungal endophyte *Epichloë gansuensis*, were inoculated with the biotrophic fungal pathogen *Blumeria graminis*. We measured the status of plant defences (associated with SA and JA signalling pathways) and the levels of resistance to the pathogen. Plants associated with the endophyte showed less disease symptoms caused by the biotrophic pathogen than plants without the endophyte. In agreement with our hypothesis, the *Epichloë* endophyte increased the plant production of SA and enhanced the expression levels of plant genes of synthesis and response to the SA hormone. The elevated expression of SA-related genes coding for putative plant enzymes with anti-fungal activities promoted by the endophyte may explain the enhanced resistance to the pathogen. The present study highlights that interaction between the plant immune system and *Epichloë* fungal endophytes can contribute significantly to the resistance of endophyte-symbiotic plants against pathogens.

## 1. Introduction

In natural environments, plants are challenged by a vast range of pathogenic microbes that compromise plant fitness [1]. Plants protect themselves from these pathogens and other plant consumers by sophisticated immune responses governed by hormonal signalling pathways, including salicylic acid (SA) and jasmonic acid (JA) [2]. The predominant model states that, whereas plant SA-dependent defence responses usually control biotrophic pathogens, JA-dependent responses confer protection against necrotrophic pathogens and insect herbivores [3,4,5]. The plant association with certain beneficial symbiotic microorganisms can complement plant defences and even promote these defences (i.e., SA and JA-dependent responses) [6,7]. This is the case of plants forming associations with *Epichloë* fungal endophytes. These fungal symbionts complement host plant defences by producing bioactive alkaloids that confer protection against a broad range of vertebrate and invertebrate herbivores [8,9]. Additionally, it has been recently reported that *Epichloë* symbionts can also induce the activation of plant SA and/or JA signalling pathways [10,11]. The activation of these plant defensive signalling pathways by beneficial symbionts can sometimes increase the levels of resistance of plants against certain attackers, including pathogens [6,11,12].

The regulation of plant defence responses by beneficial symbionts would be a consequence of the capacity of these symbionts to alter levels of key enzymes associated with SA and JA signalling pathways, including enzymes involved in the biosynthesis, regulation and/or response to these hormones [6,12]. The SA hormone is mainly synthesised in the chloroplast from chorismate and phenylalanine via isochorismate synthase (ICS) and phenylalanine ammonia-lyase (PAL) enzymes, respectively [13]. In addition, this hormone can be also produced in the cytosol from the volatile methyl salicylate (MeSA) [14]. The protein NONEXPRESSOR OF *PR* GENES 1 (NPR1) is a central regulator of SA-induced responses. In the presence of high SA levels, the redox status of cells is altered transforming inactive NPR1 oligomers into active monomers. These NRP1 monomers are transported from the cytosol into the nucleus, where they interact and co-activate TGA transcription factors (TGA = TGACG sequence-specific binding protein). These transcription factors regulate the expression of SA-responsive genes, therefore the activation of TGA proteins by NPR1 induces SA-dependent defence responses [15]. The synthesis of the JA hormone also starts in the chloroplast from the oxygenation of linolenic acids (18:3). The derived compound from this reaction (i.e., 13-hydroperoxyoctadeca-9,11,15-trienoic acid) is subsequently metabolised by three distinct enzymes resulting in the intermediate product 12-oxophytodienoic (OPDA). This product is translocated into peroxisomes and after two enzymatic steps is converted into JA hormone. JA is then transported into the cytosol, where it can be enzymatically conjugated to amino acids to produce the active form, jasmonoyl-isolecine (JA-Ile) [5]. The perception of JA-Ile is carried out by a coreceptor formed by the ubiquitin E3 ligase Skp1-Cul1-F-box protein CORONATINE INSENSITIVE 1 (SCF^COI1^) and JASMONATE ZIM DOMAIN (JAZ) proteins. JA-Ile stimulates the specific binding of COI1 and JAZ proteins, which induces the ubiquination of JAZs by SCF^COI1^ and their subsequent degradation via 26-S proteasomes [15]. JAZs proteins are repressors of transcription factors that induce the expression of JA-responsive genes. Therefore, degradation of JAZs proteins leads to the induction of JA-dependent defences [5].

*Epichloë* fungi form endophytic associations with cool-season grasses of the sub-family Poöideae [16]. In these symbioses, all tissues are colonized by these biotrophic fungi except for roots and, in most associations, the vascular bundles are free of hyphae. Vegetative tissues of host grasses are symptomless and the hyphal growth of these symbionts, located in the intercellular spaces, is fully synchronized with that of the host plant (the number of hyphae does not increase once the tissue that they are in ceases growth) [17]. The association between these endophytes and host grasses is generally mutualistic as the plant provides an ongoing supply of food, a stable internal habitat and a method of transmission by the fungal colonisation of seeds (in most associations, these endophytes are vertically transmitted), whereas the endophyte provides protection against herbivores by the production of bioactive alkaloids [16].

In addition to the anti-herbivore defences provided by *Epichloë* endophytes to host plants, these symbionts can also confer protection against certain pathogens [18]. For example, plant disease symptoms caused by the biotrophic fungal pathogens *Blumeria graminis*, *Claviceps purpurea*, *Ustilago bullata* and *Laetisaria fuciformis* were reduced by the presence of an *Epichloë* endophyte within plants [19,20,21,22,23,24]. However, distinct from the *Epichloë* fungi-based defences against herbivores, the resistance against these and other pathogen species seems to be unrelated to the fungal production of alkaloids or other *Epichloë*-derived compounds [11,25]. The activation of host plant defence responses by *Epichloë* endophytes have been posited as a potential mechanism to explain the resistance of endophyte symbiotic plants to pathogens [18,26]. Against biotrophic pathogens, for instance, the induction of plant SA-dependent defence responses by beneficial symbionts may control the incidence of these attackers on plants. In fact, it has been documented that *Epichloë* endophytes can promote the plant production of the SA hormone and also active the SA signalling pathway [10,27,28]. The activation of the host’s SA signalling pathway has been also reported in plants associated with certain mycorrhizal fungal species [29,30]. Interestingly, this ability of plant beneficial symbionts to induce host SA-dependent defence responses can increase significantly the level of plant resistance against certain pathogens [31,32].

In the present study, we studied the level of plant resistance mediated by hormonal defences in plants associated with *Epichloë* fungal endophytes against a biotrophic pathogen. We subjected *Achnatherum inebrians* plants symbiotic and non-symbiotic with the fungal endophyte *E. gansuensis* to an inoculation with the biotrophic pathogen *B. graminis*. *A*. *inebrians*, commonly known as drunken horse grass, is a perennial bunchgrass that is becoming increasingly common in north and northwest alpine and subalpine grasslands in China [33]. Nearly all *A. inebrians* plants in these grasslands are host to an *Epichloë* endophyte [34]. Individuals of this plant species are frequently infected with *B. graminis*, a fungal pathogen that causes powdery mildew disease [34]. In the infection of leaves, germinating *B. graminis* conidia form appressoria attached to the leaf surface and appressorial penetration pegs attempt to penetrate the cuticle and wall of the underlying epidermal cells. Successful infection is characterised by the formation of intracellular haustoria within living epidermal cells of leaves. The pathogen utilises these haustoria, that invaginate the plasmalemma, to obtain nutrients and water. Following the colonisation within epidermal cells, numerous *B. graminis* conidia are formed along the surface of leaves adjacent to the infected cells [35]. This continual infection of *A. inebrians* plants by *B. graminis* via conidia and the presence of haustoria within epidermal cells is in complete contrast to the infection and hyphal association of *E. gansuensis* with *A. inebrians* plants. Infection of *A. inebrians* plants by *E. gansuensis* occurs solely at the time of seed formation and hyphae are only present in the intercellular spaces of plant tissues. In these intercellular spaces, hyphae are attached to the outer surface of plant cell walls and this attachment allows the fungal endophyte to obtain nutrients from the apoplast [36].

We predicted that the association of *A. inebrians* plants with *E. gansuensis* would increase the plant SA hormone concentration and also promote the expression of genes associated with the SA-signalling pathway, enhancing the levels of plant resistance against the biotrophic pathogen *B. graminis*. In order to test this prediction, we characterised plant defence responses to the two symbiotic fungi by quantifying the concentrations of SA and JA hormones, the expression levels of genes associated with SA and JA signalling pathways and also the expression levels of other plant genes related to defences. In addition, we assessed the resistance and tolerance of plants associated with the *Epichloë* endophyte against the pathogen by measuring the disease incidence and severity on leaves and the plant growth, respectively.

## 2. Material and Methods

### 2.1. Plant and Fungal Materials

Seeds of *A. inebrians* plants symbiotic (E+) and non-symbiotic (E−) with the endophytic fungus *E. gansuensis* were generated from a single population with 100% endophyte-infection belonging to a natural grassland located at the Sunan county in the Gansu province of China. Seed were collected in 2011. These seed-source plants from this grass population had been exposed to identical environmental conditions. The presence of endophytes on these seeds was checked by looking for fungal hyphae in individual seeds stained with aniline blue using a light microscope at 40X power (following the seed squash technique) [37]. These seeds were divided into two parts; before sowing, one seed lot was treated with thiophanate-methyl fungicide to eliminate the ability of the endophyte to infect seedlings and the other lot did not receive any treatment. Two hundred seedlings of each of the two parts were sown in an experimental field area at the College of Pasture Agriculture Science and Technology of Lanzhou University in 2012. Ripe seeds produced on each plot were harvested and the endophyte presence in seedlings was evaluated in leaf sheath pieces and in seed samples treated with aniline blue, both by microscopic examination. The frequency of fungal endophytes was contrasting between treated and non-treated seed lots (i.e., 0 and 100%, respectively). These plants were individually labelled as E− and E+, respectively. These seeds were stored at 4 °C until experimentation. Conidia of the biotrophic pathogen *B. graminis* utilised in the present study were obtained from leaves of infected *A. inebrians* plants located in a greenhouse at the College of Pastoral Agriculture Science and Technology of Lanzhou University. A soft brush was used to dislodge conidia off plant leaves and the fungal material was transferred into 1.5 mL Eppendorf tubes and stored at 4 °C. Conidia were resuspended in distilled water and the suspension was examined by light microscopy at 40X power. The suspension contained only one fungal spore type and the shape and size of these conidia were consistent with *B. graminis*. 

An experiment was conducted to test the defence responses of *Epichloë* endophyte-symbiotic and non-symbiotic plants against the *B. graminis* fungus. In June 2018, seeds of E+ and E− biotypes were sown into 300 mL plastic pots (100 pots for E+ and 100 pots for E− plants) filled with 200 g of sterilised vermiculite (vermiculite was treated at 120 °C for 5 h). Each pot initially contained three seeds of the same endophyte-biotype, but only one plant was selected and maintained in each pot for the inoculation treatment (see below). The endophyte-status of each plant was confirmed by microscopic examination of the outermost leaf-sheath [37,38]. Plants were grown in a greenhouse with controlled conditions (temperature: 25 ± 2 °C; humidity: 46 ± 2%). 

The experiment was conducted following a 2 × 2 full factorial design, with the *Epichloë* endophyte plant status (E+ and E−) and the plant pathogen inoculation (P+ and P−, inoculated and non-inoculated with the pathogen, respectively) as main factors. Two-month-old E+ and E− plants were inoculated with *B. graminis*. The inoculation was performed by spraying the leaves of 50 E+ and 50 E− plants with 5 mL of a suspension of *B. graminis* containing 2 × 10^6^ spores per mL; the leaves of the other 50 E+ and 50 E− plants were treated with 5 mL of sterilised water (P+ and P− plants, respectively). After the inoculation, P+ and P− plants were maintained separately in two independent greenhouses with identical conditions for growing the experimental plants. Plants were monitored for four weeks after the pathogen inoculation.

### 2.2. Measurement Protocols

#### 2.2.1. Determination of Jasmonic Acid and Salicylic Acid Hormone Concentrations

The SA and JA hormone concentrations were quantified in leaves of E+ and E− plants at four weeks after the pathogen inoculation by means of enzyme-linked immunosorbent assays (ELISA). For this purpose, leaf samples from four E+ and three E− plants inoculated and non-inoculated with the pathogen were harvested (plants were chosen randomly from the initial plant pool), immediately frozen in liquid nitrogen and then freeze-dried for 48 h. ELISA has been often used to measure plant hormones due to it being easy to perform and has high sensitivity for detecting these compounds [11,39,40]. ELISA kits for specific detection of SA and JA hormones were used (FANKEL industrial Co. Ltd., Shanghai, China). Freeze-dried samples (=0.5 g) were ground with ball mills, then added into 5 mL of phosphate buffered saline solution (PBS, pH 7.4) and centrifuged at 3000 rpm for 20 min. A volume of 10 μL of the supernatant were diluted with 40 μL of PBS solution and this total solution (i.e., 50 μL) was transferred into a microtiter plate. One hundred microliters of specific antibodies for detection of SA and JA hormones conjugated with horseradish peroxidase enzymes was added and the solution was incubated for 60 min at 37 °C. After the incubation, the plate was washed five times with washing buffer and dried, then 50 μL of a chromogen solution (i.e., A) and the same volume of another chromogen solution (i.e., B) were added. The plate was incubated in the dark for 15 min at 37 °C. The reaction was inhibited by using 50 μL of stop solution. The absorbance was read at 450 nm with a microplate reader (DR6000, Hach, Loveland, CO, USA). Hormone concentrations were calculated using calibration curves built in each microtiter plate using the SA and JA standards provided in the kits.

#### 2.2.2. Disease Investigation 

Levels of disease caused by *B. graminis* on plants were evaluated at weeks one, two and three after the pathogen inoculation on nine E+ and nine E− inoculated plants chosen randomly from the initial pool. Plants non-inoculated with the pathogen did not show any signs of disease symptoms. The disease evaluation consisted in determining in each plant the percentage of leaves infected by the pathogen (i.e., disease incidence) and the total area of leaf tissues damaged by the pathogen (i.e., disease severity). Disease severity was calculated using the equation presented in Huang et al. [41]. 

#### 2.2.3. Determination of Plant Growth

Plant growth variables were evaluated at three weeks after the pathogen inoculation on nine plants chosen randomly from each treatment combination. The measured variables were foliar plant biomass, plant height and tiller number. The foliar plant biomass was determined by weighting fresh and dry leaf tissues. Dry tissues were obtained by treating plants at 80 °C for two days in an oven.

### 2.3. Characterisation of Plant Transcriptomes 

#### 2.3.1. Plant RNA Extraction and Library Preparation 

The plant transcriptomes were characterised by sequencing total RNAs on leaves of E+ and E- plants at four weeks after the pathogen inoculation by RNA-Seq. For this, leaf samples from three E+ and three E− plants inoculated and non-inoculated with the pathogen were harvested (plants were chosen randomly from the initial plant pool), immediately frozen in liquid nitrogen and maintained at −80 °C until analysis. The RNA extraction was performed on 100 mg of leaf samples (fresh weight) using the TRIzol reagent (Invitrogen, Carlsbad, CA, USA) (samples were not pooled at all). The extracted RNA was cleaned with the RNeasy Plant Mini Kit (Qiagen, Valencia, CA, USA) and DNA was digested using RNase-free DNase (Qiagen, Frederick, MD, USA). The RNA integrity was assessed by inspecting 1% agarose gel bands and by evaluating RNA integrity numbers (RINs) obtained from RNA Nano 6000 assays (Agilent Bioanalyzer 2100 system, (Agilent Technologies, Palo Alto, CA, USA)). RNA purities and concentrations were checked with NanoPhotometer^®^ spectrophotometers (IMPLEN, CA, USA) and Qubit^®^2.0 fluorometers (Life Technologies, CA, USA), respectively. The mRNA was purified from 3 μg of total RNA using poly-T oligo-attached magnetic beads. cDNA strands were synthesised using random hexamer primers, M-MuLV reverse transcriptase (RNase H-) and DNA Polymerase I. NEBNext adaptors were ligated to double-stranded cDNA fragments. DNA fragments were amplified by PCR, using Phusion high-fidelity DNA polymerases (New England Biolabs, Ipswich, MA, USA) and purified with the AMPure XP system (Beckman Coulter, Brea, CA, USA). Transcriptome libraries were generated using the NEBNext^®^Ultra™ RNA Library Prep Kit for Illumina^®^ (NEB, Ipswich, MA, USA) and sequenced by the Illumina HiSeq 2000 platform. The sequencing produced 6.3–11.2 billion bp pair-ended reads per sample.

#### 2.3.2. Plant Transcriptome Assembly, Annotation, Calculation of Gene Expression Values and Identification of Genes Associated with Plant Defences

High quality clean reads (=90.54 Gb) were obtained from raw reads by removing sequences containing adaptors, poly-N and low quality sequences. The TRINITY program (v. r2012-06-08) was used for the de novo transcriptome assembly of high-quality clean reads into unigenes [42]. A total of 122,923 unigenes (N50 of 1548 base pair (bp)) were obtained, with a mean length of 825.65 bp. There were 18,277 transcripts longer than 1000 bp (23.16%). The total unigene sequences (in FASTQ format) were used as queries for searching putative gene functions in several databases, namely, NR (NCBI non-redundant protein sequences), Pfam (Protein family), KOG/COG/eggNOG (Clusters of Orthologous Groups of proteins), Swiss-Prot (a manually annotated and reviewed protein sequence database), KEGG (Kyoto Encyclopedia of Genes and Genomes) and GO (gene Ontology). The annotated unigenes were 53,739. The RNA-Seq reads were mapped against the annotated unigenes in order to calculate the number of reads per annotated unigene. The expression level of each annotated gene was normalised based on the library size and the transcript lengths. These normalised gene expression levels were reported as fragments per kilobase of transcripts per million of reads (FPKM).

We were interested in characterising the overall defence response of plants, in terms of gene expression, to the presence of the *Epichloë* endophyte and *B. graminis* pathogen. For this purpose, we collected differentially expressed genes (DEGs) related to plant defences from our transcriptomic data. Plant defence-related genes were identified using GO categories (or ‘terms’) associated with this plant function, such as ‘defence response (GO: 0006952), ‘defence response to bacterium (GO: 0042742)’, ‘jasmonic acid biosynthesis process (GO: 0009695)’ and ‘response to salicylic acid (GO: 0029751)’ (see in Table S1 the complete list of defence-related GO categories and genes). Furthermore, we were also interested in describing the responses of the plant SA and JA signalling pathways to the *Epichloë* endophyte and *B. graminis* pathogen. In this analysis, we collected plant genes associated with the SA and JA signalling pathways from our transcriptomic data. These genes were identified using the reference hormonal signalling pathways depicted in Martel et al.’s [43] and Zhang et al.’s [44] papers and the SA/JA-related genes published in comprehensive reviews about the topic [14,45] (see the complete list of SA- and JA-related genes in Table S2). The RNA-seq and resequence data used in this study were deposited in the Sequence Read Achieve (SRA) of the NCBI database under accession number PRJNA748183.

#### 2.3.3. Validation of RNA-Seq Data

To validate the findings from the RNA-Seq data, expression levels of 17 randomly selected plant genes were quantified by quantitative real-time PCRs (qRT-PCR) (see the gene list and primers in Table S3). For these gene quantifications, we utilised the same RNAs that were used for characterising leaf-plant transcriptomes. cDNAs for qRT-PCR were synthesized from 2.5 µg of total RNA using MMLV reverse transcriptase (TaKaRa, Dalian, China). The RT-qPCR assays were performed using the SYBR Premix Ex Taq II Kit (TaKaRa, Dalian, China) on a 7500 Fast Real-time PCR system (Applied Biosystems, Foster City, CA, USA). Three technical replicates were carried out per sample. The relative gene expression levels were calculated using the 2−△△Ct method [46].

### 2.4. Statistical Analyses

The effects of the plant symbiotic status and the pathogen inoculation on the concentration of the SA and JA plant hormones and plant growth variables (i.e., leaf weight (fresh and dry), plant height and tillers number) were analysed with linear effects models, using the function *gls* from the *nlme* package in the R software (assuming normal distribution of errors) [47,48]. These models included the plant symbiotic status (E+ and E−) and the pathogen inoculation (P+ and P−) as categorical factors. We used the function VarIdent on the plant symbiotic status treatment for accommodating deviations in homogeneity variance in variables of the SA and JA concentrations and leaf fresh weight [49]. ANOVA assumptions were met in all these variables.

The effects of the plant symbiotic status and the experimental time on the pathogen disease incidence and severity were analysed with linear mixed models, using the function *lme* from the *nlme* package in the R software (assuming normal distribution of errors) [47]. These models included the plant symbiotic status (E+ and E−) and time (at one, two and three weeks after the inoculation of the pathogen on plants) as categorical factors and the random effect included the time nested in plant pots. Time autocorrelations were not observed between the repeated measurements in both response variables (i.e., disease incidence and severity). ANOVA assumptions were also met in these variables. 

To identify DEGs associated with the effects of the *Epichloë* endophyte on plants and *B. graminis* pathogen on E+ and E− plants, gene expression values were analysed using the *DESeq2* package in the R software (assuming negative binomial distribution) [50]. For this purpose, we first compared the expression levels of individual genes (i.e., FPKM values) calculated for E+P−, E−P+ and E+P+ plants with those obtained in E−P− plants to estimate the level of fold change (FC) of each gene per comparison. Additionally, we performed Fisher’s exact tests in order to identify individual genes with significant differences in expression levels associated with these comparisons. The probability values associated with these comparisons were adjusted for multiple testing by the Benjamini–Hochberg false discovery rate (FDR) [51]. DEGs were assigned to unigenes with FC gene expression values greater than or equal to two and with adjusted *p*-values (FDR value) less than 0.05. 

To validate the RNA-Seq data, the relationship between expression values of genes quantified by RNA-Seq and qRT-PCR was determined with linear effects models, using the function *gls* of the *nlme* package in the R software (assuming normal distribution of errors) [47,48]. The model considered gene expression values calculated by RNA-Seq as response variable and those determined by qRT-PCR as continuous covariates. ANOVA assumptions were met in this analysis. We performed post-hoc analyses on treatments when significant interactions were detected using the package *lsmeans* in R [52]. All values presented in the result section correspond to means ± standard errors (SEM).

## 3. Results

### 3.1. SA and JA Content

**Figure 1:**
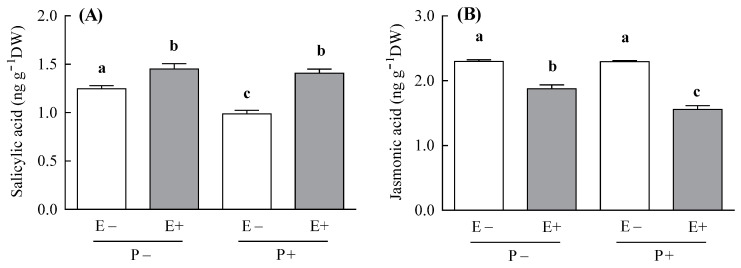
Concentration of the salicylic acid (panel **A**) and jasmonic acid (panel **B**) hormones of *Achnatherum inebrians* plants with (E+, shaded bars) and without (E−, unshaded bars) the fungal endophyte *Epichloë gansuensis* and inoculated (P+) and non-inoculated (P−) with the pathogen *Blumeria graminis*. Concentrations were measured at four weeks after the pathogen inoculation of plants. Different letters indicate significant differences at *p* < 0.05. Bars represent means values ± SEM (*n* = 3–4).

In agreement with our hypothesis, the endophyte increased the plant SA concentration, but the magnitude of this SA increment depended on the pathogen inoculation treatment (i.e., symbiosis × pathogen inoculation; Table 1). The endophyte increased the SA concentration two-fold more in plants inoculated with the pathogen, compared with the non-inoculated ones (the endophyte-mediated increment of SA was 30% and 14% for P+ and P− plants, respectively; Figure 1A). This magnitude of difference in the SA concentration was explained in part by the negative effect of the pathogen on the levels of this hormone in endophyte-free plants (i.e., the SA concentration of E− plants was decreased by 20% by the pathogen) (Figure 1A). 

Contrary to the SA, the endophyte reduced the plant concentration of JA and this JA reduction was also dependent on the pathogen inoculation treatment (i.e., symbiosis × pathogen inoculation; Table 1). The JA concentration was reduced two-fold more when endophyte-symbiotic plants were inoculated with the pathogen compared with the non-inoculated plants (the endophyte-mediated reduction of JA was 32% and 18% for P+ and P− plants, respectively; Figure 1B).

### 3.2. Pathogen Disease Incidence and Severity on Plants

**Figure 2:**
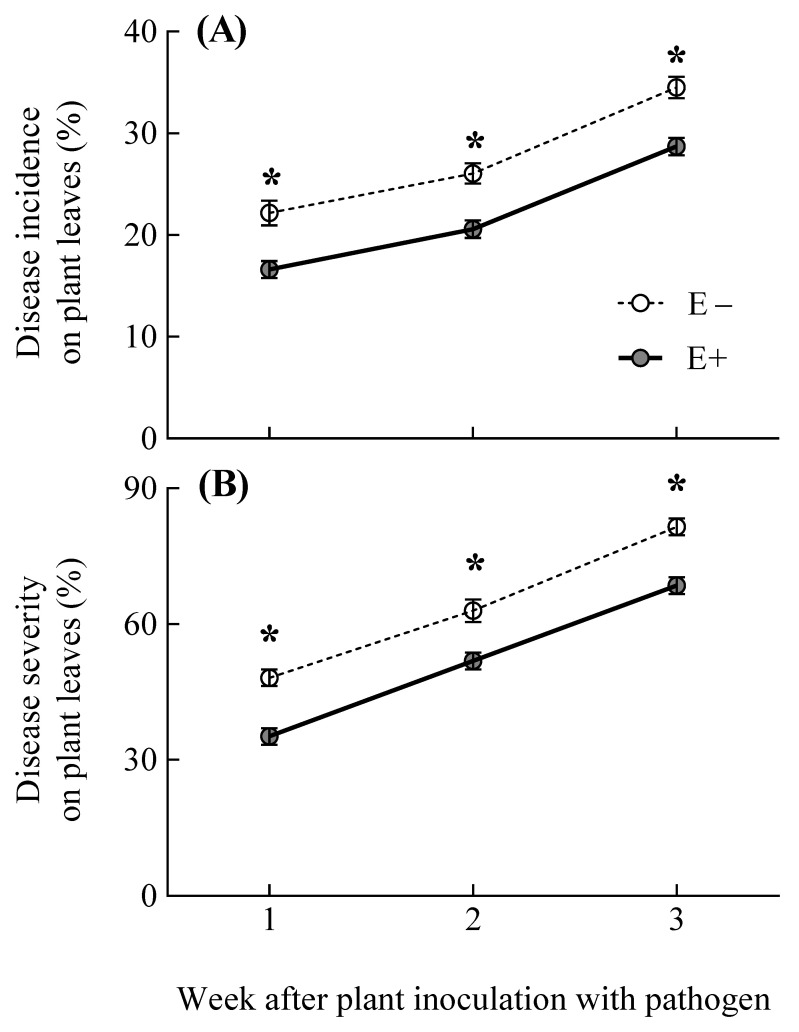
Disease incidence and severity (panels **A** and **B**, respectively) of the pathogen *Blumeria graminis* on leaves of *Achnatherum* *inebrians* plants with (E+, shaded circles and solid line) and without (E−, unshaded circles and dashed line) the fungal endophyte *Epichloë gansuensis* along the experimental time. Asterisks indicate significant differences at *p* < 0.05 between E+ and E− plants in a particular time. Circles represent means values ± SEM (*n* = 9).

In line with the endophyte induction of plant SA levels, the *Epichloë* presence in plants increased the resistance to the pathogen independently from the experimental time (i.e., symbiosis treatment and symbiosis x time for disease incidence and severity variables; Table 2) (Figure 2A,B). The proportion of plant leaves infected with the pathogen *B. graminis* was reduced by 20%, due to the plant association with *E. gansuensis* (i.e., the disease incidence on E+ and E− plant leaves was, on average, 21.27 ± 0.01% and 27.58 ± 0.01% respectively) (Figure 2A). Moreover, the severity of the pathogen disease in foliar plant tissues was decreased by 19%, due to the endophyte presence (i.e., the disease severity on E+ and E− plant leaves was, on average, 51.58 ± 2.91% and 64.20 ± 2.86% respectively) (Figure 2B). As expected, either the proportion of leaves infected by the pathogen and the severity of the disease in foliar plant tissues increased with the experimental time (i.e., time treatment; Table 2) (Figure 2A,B).

### 3.3. Plant Growth Parameters

**Figure 3:**
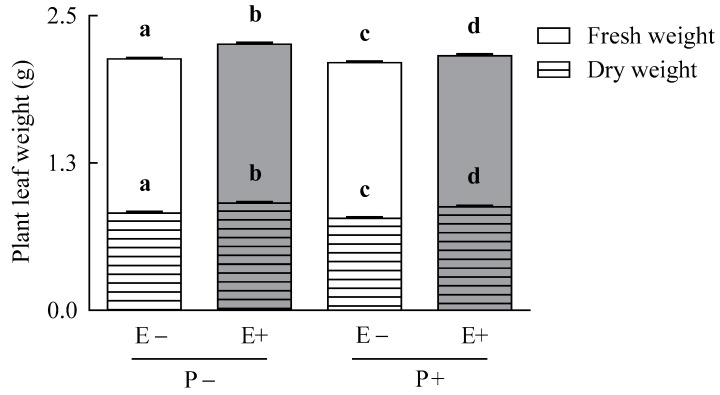
Plant leaf fresh (plain bars) and dry (stripped bars) weights of *Achnatherum* *inebrians* plants with (E+, shaded bars) and without (E−, unshaded bars) the fungal endophyte *Epichloë gansuensis* and inoculated (P+) and non-inoculated (P−) with the pathogen *Blumeria graminis*. Plant weights were measured at three weeks after the pathogen inoculation of plants. Different letters indicate significant differences at *p* < 0.05. Bars represent means values ± SEM (*n* = 9).

The endophyte fungus enhanced the foliar weight of their host plants despite of the pathogen presence (symbiosis treatment for foliar plant weight variables; Table 1) (Figure 3). On average, plant fresh and dry foliar biomass increased by 5% and 10%, respectively, due to the endophyte presence (fresh weight: 2.21 ± 0.01 and 2.11 ± 0.01 g for E+ and E− plants, respectively; dry weight: 0.89 ± 0.01 g and 0.80 ± 0.01 g for E+ and E− plants, respectively) (Figure 3). The pathogen affected negatively the height and tillers number of plants (Table 1). The pathogen infection reduced by 4% and 8% the foliar height and numbers of tiller of *A. inebrians* plants, respectively (plant height, 37.68 ± 0.17 cm and 39.46 ± 0.19 cm, for P+ and P− plants; tiller number, 9.83 ± 0.23 and 10.61 ± 0.21 # plants^−1^, for P+ and P− plants, respectively) (Table 1).

### 3.4. Characterisation of Plant DEGs Associated with Defence Responses

**Figure 4:**
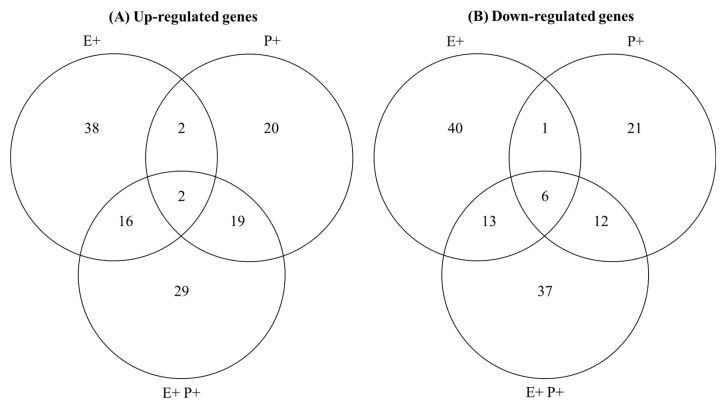
Number of up-regulated (panel **A**) and down-regulated (panel **B**) plant genes associated with defence responses given the presence of the endophyte *Epichloë gansuensis* (E+), the pathogen *Blumeria graminis* (P+) and both microorganisms (E+P+) on *Achnatherum* *inebrians* plants. The gene expression levels were measured at four weeks after the pathogen inoculation of plants (*n* = 3). The entire list of genes is presented in Table S1.

We retrieved a total of 256 unique plant DEGs associated with defence responses (see Table S1 and Figure 4). Plants associated with the *Epichloë* fungal endophyte that were inoculated with the pathogen presented the highest variation in plant genes associated with defences (i.e., E+P+ plants had 134 defence associated DEGs > E+ > P+ plant conditions) (Figure 4). The most dissimilar plant defence responses, in terms of DEGs, were between E+ and P+ plant conditions (i.e., only 11 DEGs were shared between these conditions, while 37 and 39 were shared between E+/E+P+ and P+/E+P+ conditions, respectively) (Figure 4). The expression levels of the selected genes determined by qRT-PCR were comparable with those obtained by RNA-Seq (see Figures S1 and S2).

### 3.5. Expression of Genes Associated with SA and JA Signalling Pathways

**Figure 5:**
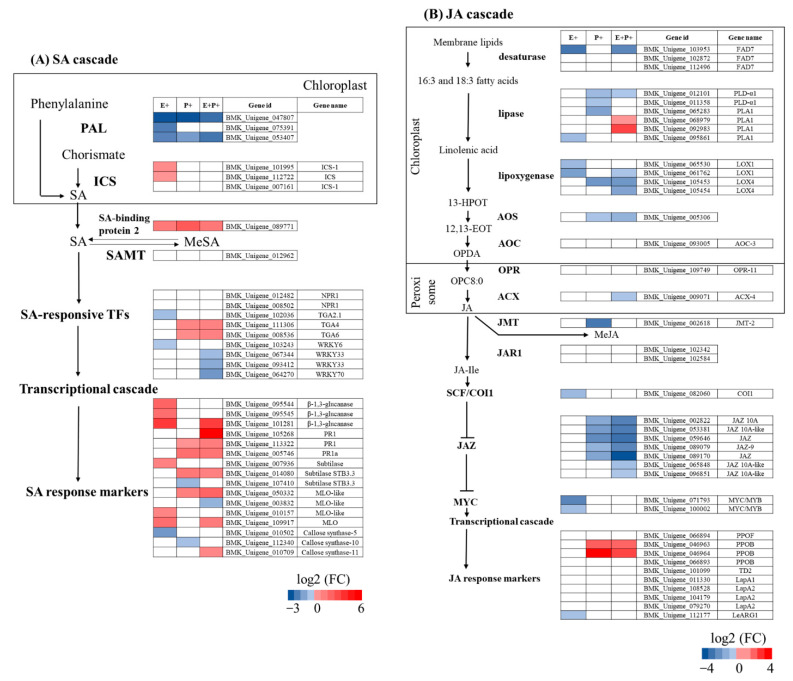
Changes in expression levels of plant genes associated with the salicylic acid (SA, panel **A**) and jasmonic acid (JA, panel **B**) signalling pathways given the presence of the fungal endophyte *Epichloë gansuensis* (E+), the fungal pathogen *Blumeria graminis* (P+) and both fungi (E+P+) on *Achnatherum* *inebrians* plants. The gene expression levels were measured at four weeks after the pathogen inoculation of plants. Proteins and enzymes are shown in bold. Arrows indicate positive regulation and truncated connectors indicate the opposite. Abbreviations: PAL, phenylalanine ammonia lyase; ICS, isochorismate synthase; MeSA, methyl salicylate; SAMT, salicylate-O-methyl transferase; NPR1, nonexpressor of PR genes 1; TGA, TGACG sequence-specific binding protein; WRKY, (T)TGAC(C/T) sequence-specific binding protein; PR1, pathogenesis-related protein 1; FAD, omega-3 fatty acid desaturase; PLD, phospholipase D; PLA1, plastochron1; LOX, lipoxygenase; 13-HPOT, 13-hydroperoxyoctadeca-9,11,15-trienoic acid; AOS, allene oxide synthase; 12,13-EOT, 12,13-epoxyoctadeca-9,11,15-trienoic acid; AOC, allene oxide cyclase; OPDA, 12-oxo-phytodienoic acid; OPR, oxophytodienoic acid reductase; OPC8:0, 3-oxo-2-(cis-2’-pentenyl)cyclopentane-1-octanoic acid; ACX, acyl coenzyme-A oxidase; OPC, OPC-8:0 CoA ligase1; JMT, jasmonic acid carboxyl methyltransferase; MeJA, methyl jasmonate; JAR1, jasmonoyl--L-amino acid synthetase JAR1; JA-Ile, JA-isoleucine conjugate; COI1, coronatine-insensitive protein 1; JAZ, jasmonate ZIM domain; MYC2, bHLH transcription factor; PPOF, polyphenol oxidase F, chloroplastic; PPOB, polyphenol oxidase E, chloroplastic-like; TD2, threonine dehydratase 2 biosynthetic, chloroplastic; LapA, leucine aminopeptidasee, chloroplastic; LeARG1, arginase 1. Red and blue colors on cells indicate significant differences in gene expression levels expressed as log2 fold changes (FC) (*n* = 3). FC- and *p*-values of each gene are presented in Table S2.

In agreement with the *Epichloë* fungal promotion of plant SA levels, the presence of these fungal symbionts on plants upregulated the expression of genes associated with SA biosynthesis, specifically those coding for putative ICS and SA-binding protein 2 enzymes. Moreover, the *Epichloë* fungus also upregulated the expression of several genes involved in the signalling and response to the SA hormone. Specifically, these genes encoded for putative β-1,3-glucanase, subtilase and MLO proteins (Figure 5A, E+ column). Opposite to the *Epichloë* upregulation of plant SA biosynthesis genes, the pathogen *B. graminis* globally reduced the expression of genes coding for putative PAL enzymes in E− plants (which are associated with SA accumulation). However, despite that SA biosynthesis genes were downregulated, the pathogen increased the expression of several genes coding for putative proteins involved in the signalling and response to the SA in endophyte-free plants (i.e., TGA transcription factors, PR1, subtilase and MLO proteins) (Figure 5A, P+ column). In E+ plants, the pathogen *B. graminis* also reduced the expression of PAL genes (similarly to E− plants), but the opposite occurred with the levels of the SA-binding protein 2 gene. Interestingly, the pathogen upregulated a higher number of genes associated with the SA response in E+ compared to E− plants. These genes encoded for putative β-1,3-glucanase, PR1 proteins, subtilase, MLO proteins and callose synthase enzymes (Figure 5A, E+P+ column).

In agreement with the *Epichloë* endophyte reduction of plant JA levels, the expression of genes associated with the production of the JA hormone were globally downregulated by this fungal mutualistic symbiont (i.e., genes coding for putative FAD7, PLA1 and LOX1 enzymes). In addition, the plant association with the *Epichloë* endophyte downregulated the expression of genes putatively coding for SCF^COI1^ enzymes, which are negative regulators of JAZ proteins. These proteins repress the expression of JA-defence responses. Thus, the downregulation of SCF^COI1^ enzymes by the *Epichloë* endophyte may stabilise JAZ proteins, therefore repressing the expression of genes of response to JA. In line with this, the expression of genes coding for putative proteins of response to JA and JA response markers were downregulated by the *Epichloë* fungus (i.e., MYC and LeARG1) (Figure 5B, E+ column). Similarly to the *Epichloë* endophyte, in E- plants, the pathogen *B. graminis* also downregulated the expression of several genes of JA biosynthesis (e.g., PLA1, LOX4 and AOS). However, despite this global downregulation in the expression of JA biosynthesis genes, the pathogen reduced the expression of several genes coding for putative JAZ proteins in endophyte-free plants, which suggests that plant JA-defence responses could have been activated. In fact, the expression of some plant genes related with the JA transcriptional cascade were upregulated by the pathogen in these plants (i.e., genes coding for putative PPOB proteins) (Figure 5B, P+ column). In E+ plants, the *B. graminis* pathogen downregulated the expression of several JA biosynthesis genes (i.e., FAD7, PLD-α1, LOX1, LOX4, AOS and ACX-4). The number of downregulated genes were higher in E+ compared to E− plants. In addition, the pathogen reduced the expression of several genes coding for JAZ proteins in endophyte-symbiotic plants, suggesting an activation of JA defence responses. In agreement with this, the pathogen increased the expressions of PPOB genes in these plants (Figure 5B, E+P+ column). 

## 4. Discussion

In the present study, we posited that the association of *A. inebrians* plants with the *Epichloë* fungus may confer protection against the biotrophic pathogen *B. graminis* and this protection would result from the *Epichloë* fungal induction of host plant SA-dependent defences. Our results show that, similarly to results obtained by previous studies [23], plants associated with the *E. gansuensis* endophyte show effectively less disease symptoms caused by *B. graminis* than non-symbiotic plants. In agreement with our hypothesis, the *Epichloë* endophyte increased the plant production of SA and enhanced the expression level of several genes of response to the SA hormone. Among these genes, the upregulation of genes coding for β-1,3-glucanase and callose synthase enzymes by *Epichloë* symbionts could explain, in part, the enhanced resistance to the pathogen due to their recognised anti-fungal activities [53,54]. Additionally, the presence of *E. gansuensis* enhanced the host plant biomass, which could have increased the tolerance of symbiotic plants against the pathogen.

The plant association with beneficial symbionts can induce plant defences and, sometimes, this induction enhances the plant resistance against pathogens [5,12]. In the present study, the *Epichloë* endophyte increased the concentration of the host plant SA hormone (see also Ambrose et al. [27]). Our gene expression results suggest that this enhanced SA concentration resulted from the apparent *Epichloë* fungal induction of putative plant isochorismate synthase and SA-binding protein 2 enzymes. In plants of *Arabidopsis thaliana* interacting with microorganisms, these enzymes have been reported to be dominating the SA production [14,45]. In addition to the increased plant SA levels, several plant genes of response to the SA hormone were also upregulated by the *E. gansuensis* endophyte [10]. From the point of view of the resistance against fungal pathogens, the positive effect of *Epichloë* fungi on the expression of plant genes coding for putative β-1,3-glucanase enzymes is particularly interesting. In plants associated with mycorrhizal fungi, the resistance of symbiotic plants against certain fungal pathogens is boosted when mycorrhizal symbionts increased the plant levels of β-1,3-glucanase enzymes (reviewed in Jung et al. [55]). Enhanced resistance levels to pathogens due to the accumulation of these compounds have also been reported in certain plants cultivars [56]. The plant protection against fungal pathogens conferred by these enzymes is associated with their capacity to degrade structural components of fungal cell walls (i.e., β-D-glucans). This degradation generally inhibits the hyphal growth, thus avoiding the spread of the pathogen within plant tissues [53].

The plant defence responses to the presence of the pathogenic and the mutualistic fungal symbionts were highly dissimilar. The pathogen *B. graminis* reduced plant SA concentration and downregulated the expression of part of the SA biosynthesis genes, while the opposite occurred in plants associated with the *Epichloë* mutualistic endophyte. Moreover, only 6% of plant DEGs associated with defence responses were shared between E+ and P+ plants (i.e., only 11 out of 184 total DEGs for E+ and P+ plants). This high dissimilarity in plant defence responses is expected, due to these biotrophic fungi having totally contrasting lifestyles and growing patterns within plants, as previously stated. It is likely that the *B. graminis* suppression of the plant SA hormone production was part of the strategy performed by this pathogen to facilitate the colonisation of *A. inebrians* plants. In fact, pathogens usually manipulate plant defences in order to enhance the invasion of plants [57]. For example, the active suppression of plant SA biosynthesis by the fungal pathogen *U. maydis* increased its colonisation into *Zea mays* plants [58]. However, whereas the *B. graminis* suppression of plant SA biosynthesis could have facilitated its infection of *A. inebrians* plants, several plant genes of SA response were activated by the pathogen’s presence (i.e., genes coding for putative PR1, subtilase and MLO proteins). This latter finding suggests that SA plant defence responses were, at least, partially activated by the *B. graminis* pathogen. In addition, plant JA-dependent defence responses seemed to be also induced by the pathogen (i.e., the expression of genes coding for putative JAZ proteins were upregulated by *B. graminis*). While not usually involved in defence responses against biotrophic microorganisms, it has been documented that plant JA-dependent defences can contribute to the resistance against some biotrophic pathogens [59,60]. These results collectively suggest that despite the fact that the pathogen *B. graminis* could supress the plant SA production, SA- and JA-dependent defence responses were activated, which may assist in the containment of the pathogen disease (in fact, after three weeks of the pathogen inoculation, only ca. 27% of plant leaves were infected by *B. graminis*).

The plant association with the *Epichloë* endophyte resulted in higher SA levels and more complex SA-dependent defence responses (i.e., involving a higher number of DEGs) against the pathogen *B. graminis*, compared to plants without the mutualistic symbiont. It is likely that this *Epichloë*-mediated induction of SA production was key in the increment of levels of plant resistance against *B. graminis* [18]. It is remarkable that despite the capacity of the pathogenic symbiont to regulate the plant SA production of *A. inebrians* plants (observed on non-symbiotic plants), levels of this hormone were increased when the pathogen interacted with plants associated with the *Epichloë* endophyte. This indicates that the promoting effect of the *Epichloë* endophyte on SA biosynthesis was strong enough to overcome the pathogen suppression of the SA hormone production. The defence response of plants with the mutualistic symbiont to the pathogen involved a higher number of SA-associated DEGs than endophyte-free plants. Among the upregulated SA-associated genes of plants with the mutualistic symbiont with potential roles in the resistance to fungal pathogens were those coding for putative β-1,3-glucanase and callose synthase enzymes. The increased action of β-1,3-glucanase enzymes could have inhibited the growth of *B. graminis* on plants associated with the *Epichloë* endophyte. Moreover, the putative accumulation of callose synthase enzymes within tissues of plants with the mutualistic symbiont could have also limited the pathogen dissemination. These enzymes produce callose, that is, a β-glucan polysaccharide that reinforces plant cell walls obstructing the penetration of fungal hyphae into plant tissues [54]. In fact, enhanced callose deposition in tissues of plants associated with mycorrhizal fungi acted as an effective barrier against the colonisation of certain fungal pathogens [59,60]. Similar to the response of endophyte-free plants to the pathogen *B. graminis*, JA defence responses seemed to be also activated in *Epichloë*-associated plants that interacted with the pathogen. As was already mentioned, the activation of this pathway could also have contributed to the resistance of *A. inebrians* plants against *B. graminis* [60]. In addition to resistance, tolerance is another strategy that plants present to protect themselves from pathogens [61]. In the present study, the *Epichloë* endophyte improved the growth of host plants under the pathogen presence. This enhanced plant growth may have contributed to alleviating the negative effect of the pathogen on plant fitness [62]. For example, survival of *Bromus auleticus* plants challenged by the pathogen *U. bullata* was increased by the association of plants with a growth promoting *Epichloë* endophyte species [63] (and see also Chen et al. [64]). 

## 5. Conclusions

In conclusion, the present study highlights that interaction between the plant immune system and the presence of *Epichloë* fungal endophytes can contribute significantly to the resistance of endophyte-symbiotic plants against pathogens (see also Shi et al. [11]). Our results suggest that the induction of the plant SA signalling pathway by the *E. gansuensis* endophyte would regulate the infection of plants by the biotrophic fungal pathogen *B. graminis*. The enhanced resistance to the pathogen of endophyte plants may be explained by the potential increment in the levels of plant enzymes with antifungal activities associated with the SA signalling pathway promoted by the *Epichloë* symbiont.

## Figures and Tables

**Table 1:** ANOVA table showing the effects of plant symbiosis status and pathogen inoculation on the concentration of plant hormones (salicylic acid and jasmonic acid) and plant growth parameters (fresh and dry leaf weights, height and tillers number) of *Achnatherum* *inebrians* plants with (E+) and without (E−) the fungal endophyte *Epichloë gansuensis* and inoculated (P+) and non-inoculated (P−) with the pathogen *Blumeria graminis*. Hormones and plant growth performance variables were measured at four and three weeks, respectively, after the pathogen inoculation of plants. Statistically significant effects are highlighted in bold. Different letters on plant growth performance values indicate significant differences at *p* < 0.05 (post hoc test). Replicate numbers are indicated in parenthesis. Values are mean ± SEM.

Response Variable	Treatment	*df*	F or *t*-Value	*p*-Value	E−	E+	E−	E+
					P−		P+	
Salicylic acid (ng g^−1^ DW) (*n* = 3–4)	Symbiosis	1,10	53.52	<0.001	-	-	-	-
	Pathogen	1,10	21.78	**<0.001**				
	Symbiosis × Pathogen	1,10	6.49	**0.028**				
Jasmonic acid (ng g^−1^ DW) (*n* = 3–4)	Symbiosis	1,10	172.56	**<0.001**	-	-	-	-
	Pathogen	1,10	2.28	0.161				
	Symbiosis × Pathogen	1,10	12.77	**0.005**				
Leaf fresh weight (g) (*n* = 9)	Symbiosis	1,32	110.45	**<0.001**	-	-	-	-
	Pathogen	1,32	41.78	**<0.001**				
	Symbiosis × Pathogen	1,32	15.10	**<0.001**				
Leaf dry weight (g) (*n* = 9)	Symbiosis	1,32	192.53	**<0.001**	-	-	-	-
	Pathogen	1,32	36.53	**<0.001**				
	Symbiosis × Pathogen	1,32	0.59	0.449				
Plant height (# plant ^−1^) (*n* = 9)	Symbiosis	1,32	1.69	0.100	39.11 ± 0.28a	39.81 ± 0.23a	37.38 ± 0.23b	37.97 ± 0.22b
	Pathogen	1,32	5.27	**<0.001**				
	Symbiosis × Pathogen	1,32	0.23	0.822				
Tillers number (# plant ^−1^) (*n* = 9)	Symbiosis	1,32	1.09	0.304	10.33 ± 0.33a	10.88 ± 0.26a	09.77 ± 0.36b	09.88 ± 0.30b
	Pathogen	1,32	5.93	**0.020**				
	Symbiosis × Pathogen	1,32	0.48	0.491				

**Table 2:** Disease incidence and severity of the pathogen *Blumeria graminis* on leaves of *Achnatherum* *inebrians* plants with (E+) and without (E−) the fungal endophyte *Epichloë gansuensis* along the experimental time. Statistically significant effects are highlighted in bold. Values of means, SEM, replicate numbers and post hoc statistical differences are showed at Figure 2A,B.

Response Variable	Treatments	*df*	F	*p*-Value
Disease incidence on plant leaves (%)	Symbiosis	1,16	48.85	<0.001
	Time	1,32	80.48	<0.001
	Symbiosis ×Time	1,32	0.01	0.985
Disease severity on plant leaves (%)	Symbiosis	1,16	59.25	<0.001
	Time	1,32	144.14	<0.001
	Symbiosis ×Time	1,32	0.148	0.862

## Data Availability

All data supporting the findings of this study are available within the paper and within its supplementary materials published online. The RNA-seq used in this study have been deposited in the Squence Read Achieve (SRA) of the NCBI database under accession number PRJNA748183.

## Supplementary Materials

The following are available online at https://www.mdpi.com/article/10.3390/jof7080633/s1, Figure S1. Relationship between expression values of genes quantified by qRT-PCR and RNA-Seq from *Achnatherum inebrians* plants associated with the endophyte *Epichloë gansuensis* (E+), the pathogen *Blumeria graminis* (P+) and both microorganisms (E+P+) (the gene expression levels were measured at four weeks after the pathogen inoculation on plants; the complete list of genes is described in Table S3; relative gene expression values were referred to the E-P− condition (i.e., plants free of *E. gansuensis* and *B. graminis* microorganisms) and expressed as log2 fold changes (FC) (*n* = 3)), Figure S2. DEGs (differently expressed genes) identified by RNA-Seq were validated by quantitative qRT-PCR analyses (*n* = 3) (lines indicate the relative expression level determined by qRT-PCR of each gene using the 2−ΔΔCt (cycle threshold) method (right y-axis); histograms represent FPKM (fragments per kilobase of exon model per million mapped fragments) of the differently expressed genes according to RNA-Seq (left y-axis); the complete list of genes is described on Table S3; E+ and E− indicate *Achnatherum inebrians* plants with and without the fungal endophyte *Epichloë gansuensis*, respectively; P+ and P− mean *A. inebrians* plants inoculated and non-inoculated with the pathogen *Blumeria graminis*, respectively), Table S1. Complete list of plant defence-related GO categories and genes, Table S2. Complete list of genes associated with plant SA and JA signalling pathways, Table S3. Primers used for qRT-PCR.
