# Supplementary material for: The Plant Salicylic Acid Signalling Pathway Regulates the Infection of a Biotrophic Pathogen in Grasses Associated with an Epichloë Endophyte
Source: J Fungi (Basel). 2021 Aug 4;7(8):633. doi: 10.3390/jof7080633 (PMC8399569; doi:10.3390/jof7080633)
Supplement: Supplementary file 1 [file jof-07-00633-s001.zip › Supporting_Information-2021.8.4.pdf]

SUPPORTING INFORMATION

Table S3: *Primers used for qRT-PCR.*

Gene name	#ID	Primers (5'-3')	
calcium-dependent protein kinase (CPK)	BMK_Unigene_089127	Forward	GCC GAC GCA GTT GTT TCC
		Reverse	TCC ACC GCT CGT CTC CAA TG
	BMK_Unigene_101535	Forward	CTC TGC GTA CCG TGT GAT GT
		Reverse	CAG CAC CAT TTG GGC CTT TC
pathogenesis-related protein 1 (PR1)	BMK_Unigene_005746	Forward	TCA GAC TTG TCT CCC GTG GT
		Reverse	ACC CGG AGT CTT TTC ATG GG
respiratory burst oxidase homolog protein B (RBOH)	BMK_Unigene_108053	Forward	AAC TGT CGA TCC ACT CCA CC
		Reverse	GCT TGG GGT TTT TCG CCT TG
jasmonate ZIM domain-containing protein (JAZ)	BMK_Unigene_089170	Forward	GTG AGT ACG CAC TAA CCA CG
		Reverse	GCA AAG GCT TGT ACA GGG GA
ethylene-insensitive protein 3 (EIN3)	BMK_Unigene_009272	Forward	GCA CCA GTC GTA CCA GAA GG
		Reverse	GAG CGA CGC TAA CCT ATC CC
auxin responsive GH3 gene family (GH3)	BMK_Unigene_104945	Forward	GGT TCT TGA ACT GCT CGC TC
		Reverse	GGG GCT CTA CTT CCT GTT CG
nitric-oxide synthase (NOS)	BMK_Unigene_010760	Forward	GCC ACC TAC GAC CTG AAG AA
		Reverse	CGA CAA CAG CTT GCA CCT TC
disease resistance protein RPP13-like (PRM1)	BMK_Unigene_071076	Forward	GTC AGC CAG TCG TTG CTT TG
		Reverse	GCG ACG TCT CCT ACG ACA TT

probable WRKY transcription factor 2 (WRKY 1/2)	BMK_Unigene_086590	Forward	AGC AAC AAG GTA AGG GGT GT
		Reverse	GGA CGG ACA GAC ATA CAA CCT
	BMK_Unigene_066637	Forward	TTC TTT CCC TCC GGC GAA C
		Reverse	TCT TCG CCG TTC GTT CGT T
SAUR family protein (SAUR)	BMK_Unigene_085384	Forward	CAT GGC CCG GCT TAG TTC TT
		Reverse	AGT CAG CAA GCC GTT TCA GG
	BMK_Unigene_087406	Forward	GAT CAG TTC TTG GAG AGG CGG
		Reverse	GGA GTT CGG GTA CAA GCA GG
two-component response regulator ARR-A family (A-ARR)	BMK_Unigene_095193	Forward	CAC CTT CCT GTC GCT CTT CG
		Reverse	TTC GTC TCG TTG TTT GGG CT
phytochrome-interacting factor 4 (PIF4)	BMK_Unigene_104592	Forward	GGT GAT GCA TCC GTT CCT CT
		Reverse	CGT CCA TCG TTG TCG GAT CT
auxin-responsive protein IAA (AUX/IAA)	BMK_Unigene_094098	Forward	ACA TGC GCC ACC GTT TAA AAT
		Reverse	GCT GTT TGT TGT GTG GAG CA
Nonexpressor of PR genes 1 (NPR1)	BMK_Unigene_092391	Forward	AAC GGT TGC CCG TTG TAT CT
		Reverse	ACC TCG AAA ACC GAG TTG CT
TGACG-sequence-specific DNA-binding protein TGA-2.1 (TGA)	BMK_Unigene_102036	Forward	TTC CTG CTG CAA AGA GTG GT
		Reverse	TTC TGG CCT GCA GTA CCT TG
serine/threonine-protein kinase SRK2 (SnRK2)	BMK_Unigene_103278	Forward	AAG AAC TTC CGG CGC TAT GT
		Reverse	GGG TCA GCT ACT GCC ATT CC
	BMK_Unigene_091577	Forward	GCA GTC TTG GGA TAC GTG GA
		Reverse	CTG GTC GGT GGT TAC CCT TT

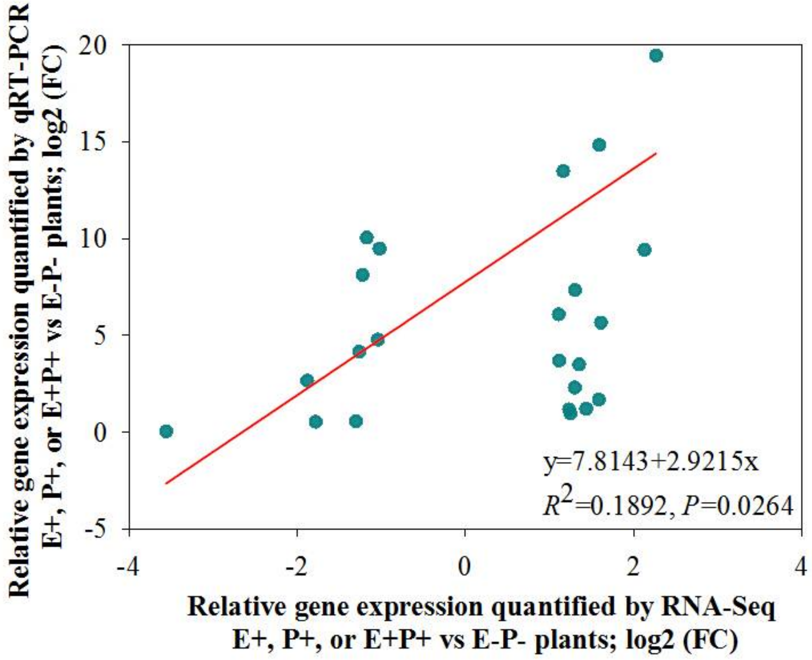

Figure S1: *Relationship between expression values of genes quantified by qRT-PCR and RNA-Seq from Achnatherun inebrians plants associated with the endophyte Epichloë gansuensis (E+), the pathogen Blumeria graminis (P+), and both microorganisms (E+P+). The gene expression levels were measured 31/2 weeks after the pathogen inoculation on plants. The complete list of genes is described on Table S3. Relative gene expression values were referred to the E−P− condition (i.e. plants free of endophytes and pathogens) and expressed as log2 fold changes (FC) (n = 3).*

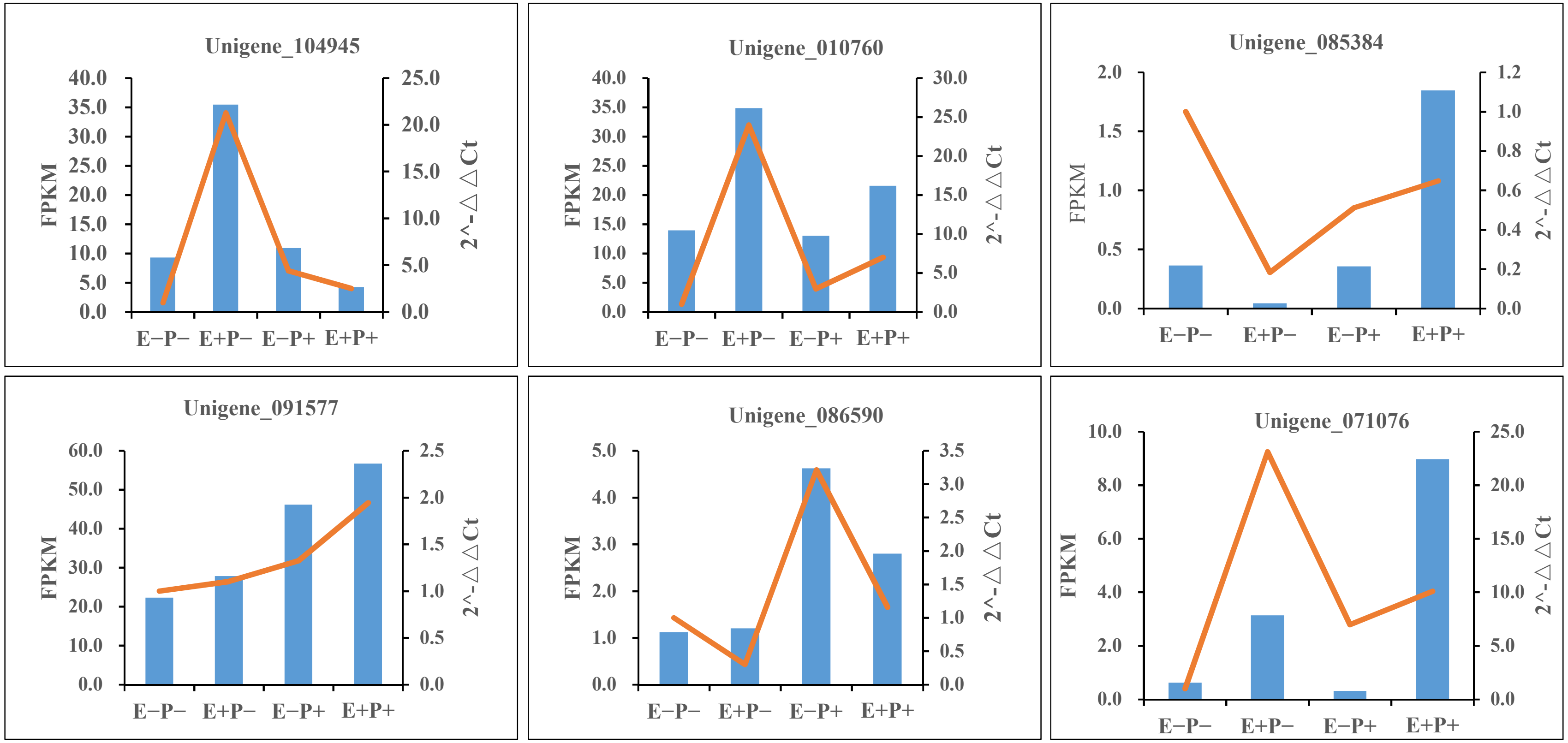
Unigene_104945
FPKM
2^-△△Ct
40.0
35.0
30.0
25.0
20.0
15.0
10.0
5.0
0.0
25.0
20.0
15.0
10.0
5.0
0.0
E−P− E+P− E−P+ E+P+
Unigene_010760
FPKM
2^-△△Ct
40.0
35.0
30.0
25.0
20.0
15.0
10.0
5.0
0.0
30.0
25.0
20.0
15.0
10.0
5.0
0.0
E−P− E+P− E−P+ E+P+
Unigene_085384
FPKM
2^-△△Ct
2.0
1.5
1.0
0.5
0.0
1.2
1.0
0.8
0.6
0.4
0.2
0.0
E−P− E+P− E−P+ E+P+
Unigene_091577
FPKM
2^-△△Ct
60.0
50.0
40.0
30.0
20.0
10.0
0.0
2.5
2.0
1.5
1.0
0.5
0.0
E−P− E+P− E−P+ E+P+
Unigene_086590
FPKM
2^-△△Ct
5.0
4.0
3.0
2.0
1.0
0.0
3.5
3.0
2.5
2.0
1.5
1.0
0.5
0.0
E−P− E+P− E−P+ E+P+
Unigene_071076
FPKM
2^-△△Ct
10.0
8.0
6.0
4.0
2.0
0.0
25.0
20.0
15.0
10.0
5.0
0.0
E−P− E+P− E−P+ E+P+

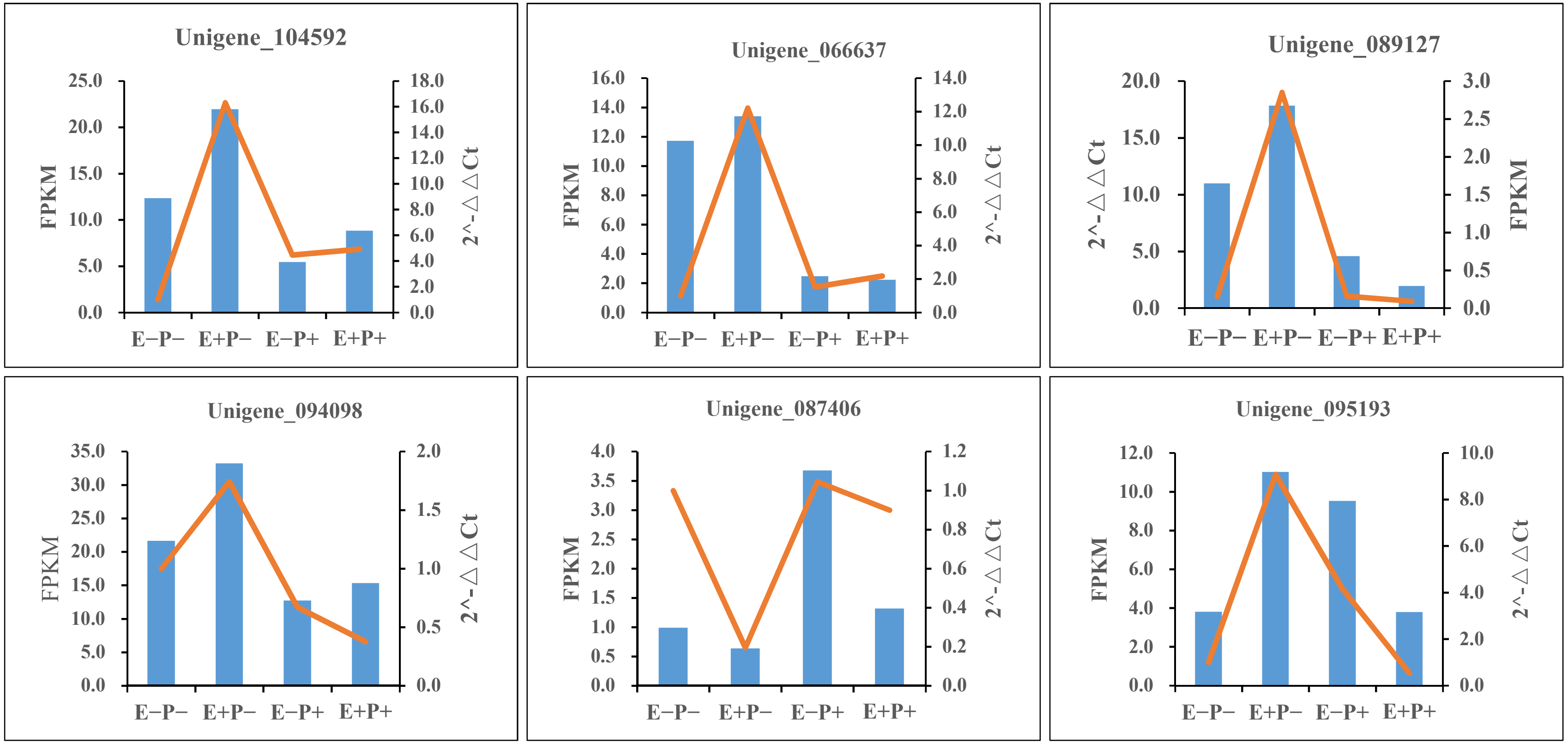

Figure S2: *DEGs (differently expressed genes) identified by RNA-Seq were validated by quantitative qRT-PCR analyses. Histograms indicate the relative expression level determined by qRT-PCR of each gene using the 2−ΔΔCt (cycle threshold) method (left y-axis). Lines represent FPKM*

(frag-ments per kilobase of exon model per million mapped fragments) of the differently expressed genes according to RNA-Seq (right y-axis). E+ and E− indicate Achnatherum inebrians plants with and without the fungal endophyte Epichloë gansuensis, respectively. P+ and P− mean A. inebrians plants inoculated and non-inoculated with the pathogen Blumeria graminis, respectively. The complete list of genes is described on Table S3.
